# The historical evolution of the neurological examination

**DOI:** 10.1055/s-0046-1817052

**Published:** 2026-03-12

**Authors:** Bruno Bertoli Esmanhotto, Gustavo Leite Franklin, Gabriel Albini, Helio Afonso Ghizoni Teive, Gerson Alves Pereira Junior

**Affiliations:** 1 Pontifícia Universidade Católica do Paraná, Escola de Medicina, Disciplina de Neurologia, Curitiba PR, Brazil.; 2 Universidade Federal do Paraná, Curso de Medicina, Departamento de Medicina Interna, Curitiba PR, Brazil.; 3 Universidade de São Paulo, Hospital de Reabilitação de Anomalias Craniofaciais, Pós-Graduação em Ciências da Reabilitação, Bauru SP, Brazil.

**Keywords:** Clinical Reasoning, Neurologic Examination, Evidence-Based Medicine

## Abstract

The neurological examination has evolved from rudimentary clinical observations in ancient civilizations to a structured and indispensable tool in modern medical diagnostics. Early contributions from Hippocratic medicine emphasized the brain as the seat of cognition and introduced systematic observation of neurological signs. In the 19 ^th^ century, figures such as Jean-Martin Charcot, Wilhelm Erb, William Gowers, and Joseph Babinski established the foundations of the modern neurological exam through anatomoclinical correlations, standardization of reflex assessment, and structured clinical reasoning. Throughout the 20 ^th^ century, seminal textbooks—such as those by Mills, McKendree, DeJong, and Wartenberg—helped consolidate and disseminate neurological semiology globally. More recently, evidence-based approaches, exemplified by Steven McGee's work, have introduced statistical rigor to bedside examinations. Despite advances in neuroimaging and electrophysiology, examinations remain a cornerstone of clinical neurology, guiding diagnosis, promoting cost-effective care, and reinforcing the doctor–patient relationship.

## INTRODUCTION

The neurological physical examination has evolved from ancient observational practices to a core element of clinical diagnosis. Despite technological advances, it remains vital today for guiding investigations, reducing costs, and strengthening the doctor–patient relationship. 1 Its systematization began in the late 19 ^th^ century, notably with William Gowers's “A Manual of Diseases of the Nervous System” (1886), which introduced key concepts, such as the upper motor neuron. 2

In the early 20 ^th^ century, authors such as Charles Mills and Charles McKendree advanced neurological teaching, with McKendree's 1928 “Neurological Examination” offering a structured, practical guide. 3 Robert Wartenberg's 1945 “Examination of Reflexes” and Russell DeJong's 1950 “The Neurologic Examination” further emphasized reproducibility and bedside reasoning. 4 Later, comprehensive works like “Handbook of Clinical Neurology” helped establish neurology as a global specialty. 5

In 2001, Steven McGee's “Evidence-Based Physical Diagnosis” introduced statistical tools, such as likelihood ratios, into the interpretation of neurological signs, reflecting a shift toward diagnostic precision ( Table 1 ). 6

## HISTORICAL ROOTS

Ancient Egyptian records, such as the Edwin Smith Papyrus (circa3000 BCE), offer some of the earliest evidence of structured medical thinking. They include descriptions of neurological signs following cranial trauma—an early form of clinical observation. 7

Hippocrates recognized the brain as the organ responsible for consciousness and cognition. His writings, especially “On the Sacred Disease”, include detailed accounts of epilepsy, hemiplegia, and paraplegia, illustrating early neurological reasoning. 8 In “On Injuries of the Head”, he described the assessment of skull fractures, highlighting the role of medical history and physical signs in diagnosis. 9 The “Corpus Hippocraticum” also references hemianopsia and the importance of visual field testing, marking one of the earliest known descriptions of a systematized neurological exam. 10 By combining clinical observation with anatomical reasoning, Hippocrates recognized and described conditions like apoplexy (stroke), ataxia, and loss of consciousness, anticipating what would later become the syndromic approach in neurology. 11

## THE FOUNDATIONS OF THE MODERN NEUROLOGICAL EXAMINATION

Jean-Martin Charcot was of paramount importance in establishing the neurological examination as a systematic clinical tool. His approach, known as the anatomoclinical method, correlated bedside observations with autopsy findings, allowing precise localization of neurological lesions. This led to the identification of distinct clinical syndromes, such as multiple sclerosis and amyotrophic lateral sclerosis (ALS). 12 He also employed instruments to record tremors and assess muscle strength, contributing to the standardization and objectivity of clinical assessment. 13

Jackson introduced the principles of cerebral localization and hierarchical organization of the nervous system. He laid the groundwork for interpreting focal neurological signs and understanding the clinical relevance of cortical lesions. 14 Wilhelm Erb emphasized the diagnostic value of deep tendon reflexes, coidentifying the patellar reflex and standardizing its examination. He also contributed to the classification of neuromuscular diseases and defined anatomical landmarks, such as Erb's point. 15

**Fig. 1:**
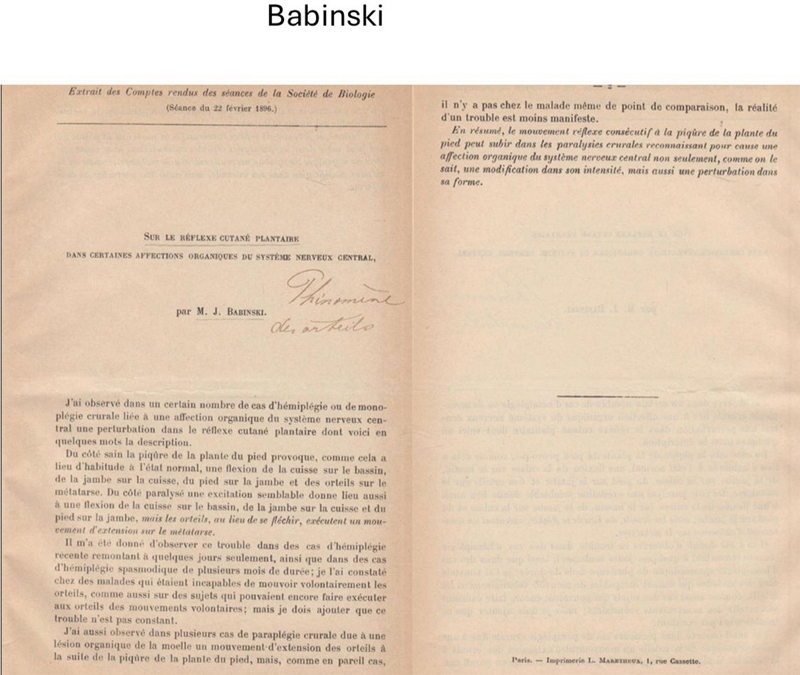
Photograph of the original paper by Babinski.

Gowers advanced the neurological examination by integrating detailed clinical observation with a structured approach. His “Manual of Diseases of the Nervous System” (1886–1899) provided comprehensive descriptions of reflexes, motor and sensory signs, and disease differentiation. He also helped standardize the use of the reflex hammer. 16 Babinski's 1896 description of the extensor plantar response (Babinski sign, Figure 1 ) allowed reliable differentiation between organic and functional paralysis. It became a cornerstone in assessing pyramidal tract dysfunction and remains one of the most specific signs in neurology. 17

## EVOLUTION OF EXAMINATION DOMAINS

Motor assessment was refined through the work of Romberg, Todd, Charcot, and Gowers. The examination of strength, tone, and motor patterns enabled lesion localization and remains central to neurological diagnostics. 18

**Fig. 2:**
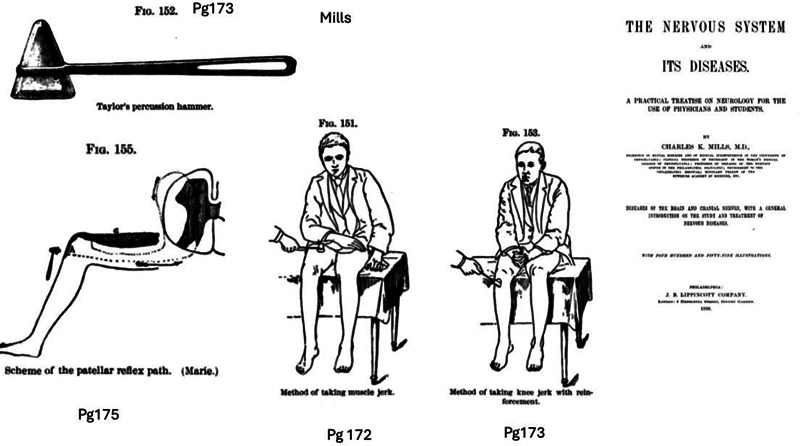
Illustrations from Charles K. Mills' “The Nervous System and Its Diseases” (1898), depicting early techniques in tendon reflex examination. From left to right: Taylor's percussion hammer (Figure 152); method of eliciting the muscle jerk (Figure 151); method of eliciting the knee jerk with reinforcement (Jendrassik maneuver; Figure 153); schematic representation of the patellar reflex arc (Marie; Figure 155).

The patellar, abdominal, and plantar reflexes were standardized in the late 19 ^th^ century, especially by Erb and Babinski, offering objective criteria for identifying pyramidal tract lesions ( Figure 2 ). 15

Based on anatomical pathways, sensory testing incorporated pain, temperature, vibration, and proprioception by the early 20 ^th^ century. Disorders like tabes dorsalis helped solidify these tests. 19

The clinical examination of cranial nerves developed with growing knowledge of brainstem anatomy. Systematic testing became standard as neurology emerged as a specialty. 20

Finally, the invention of the ophthalmoscope in 1851 revolutionized visual examination, allowing direct inspection of the optic nerve and retina. 10

## TWENTIETH-CENTURY CONSOLIDATION THROUGH TEXTBOOKS

Charles K. Mills's 1898 “The Nervous System and Its Diseases” promoted clinical integration but was soon surpassed by more specialized works. 3

Edited by Lewandowsky, then Bumke and Foerster, the “Handbuch der Neurologie” (1910–1937) systematized clinical neurology and nosology. 5 In 1928, Charles A. McKendree published one of the first manuals entirely devoted to neurological exam techniques, separating signs from pathology. 3 In 1945, Robert Wartenberg's “Examination of Reflexes” introduced standardized methods for reflex testing, many of which bear his name. 4 Russell DeJong's 1950 “The Neurologic Examination” became a foundational American textbook, emphasizing clinical reasoning and anatomical localization; it was later updated by William Campbell. 4

Furthermore, Steven McGee's 2001 “Evidence-Based Physical Diagnosis” applied statistical rigor to physical signs, using likelihood ratios and meta-analyses. It marked a paradigm shift toward diagnostic precision. 6

**Fig. 3:**
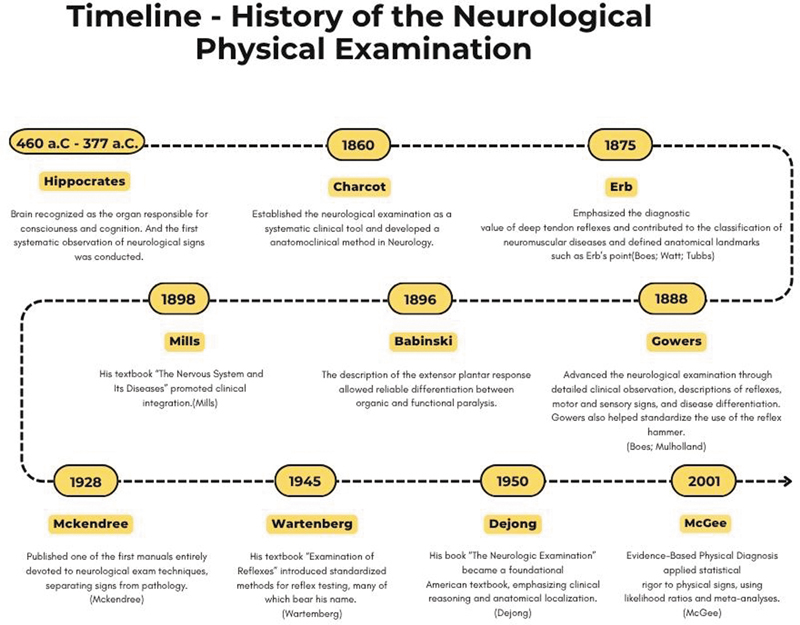
Timeline – History of the Neurological Physical Examination.

In the 20 ^th^ century, neurology was consolidated as an autonomous discipline, and the neurological examination became standardized, serving as the main diagnostic tool before the advent of complementary methods such as neuroimaging and electromyography ( Figure 3 ). Despite technological advances, the neurological examination remains irreplaceable for the localization and characterization of neurological syndromes, providing the foundation for the effective use of modern diagnostic tools. 1

In the recent years, evidence-based medicine (EBM) has introduced greater rigor into clinical semiology. The concept of likelihood ratio (LR) quantifies the diagnostic impact of a clinical finding: an LR+ >10 or an LR− <0.1 indicates a high predictive value. 21 Steven McGee, in “Evidence-Based Physical Diagnosis”, systematically analyzed clinical signs through the lens of EBM, while Scott Stern, in “Symptom to Diagnosis”, proposed a symptom-centered approach that integrates semiology, hypotheses, and investigations. 6 21

**Table 1:** Overview of contributions to the evolution of the neurological examination

Time	Important physicians and writers	Contributions
Ancient Greece	Hippocrates	Hippocratic medicine emphasized the brain was the organ responsible for consciousness and cognition. His writings include important accounts of epilepsy, hemiplegia, and paraplegia, illustrating early neurological reasoning (Panourias).
19th Century	Charcot, Erb, Gowers, Babinski	Established the foundations of the modern neurological exam through anatomoclinical correlations, standardization of reflex assessment, and structured clinical reasoning.
20th Century	Mills, McKendree, Wartenberg, and Dejong	Helped consolidate and disseminate neurological semiology globally.
21st Century	McGee	Evidence-based approaches, exemplified by Steven McGee's work, have introduced statistical rigor to bedside examination.
